# Security Considerations for E-Mental Health Interventions

**DOI:** 10.2196/jmir.1468

**Published:** 2010-12-19

**Authors:** Kylie Bennett, Anthony James Bennett, Kathleen Margaret Griffiths

**Affiliations:** ^1^e-hubCentre for Mental Health ResearchThe Australian National UniversityCanberraAustralia

**Keywords:** Mental health, health technology, Internet, implementation, confidentiality, data collection, privacy, computer security

## Abstract

Security considerations are an often overlooked and underfunded aspect of the development, delivery, and evaluation of e-mental health interventions although they are crucial to the overall success of any eHealth project. The credibility and reliability of eHealth scientific research and the service delivery of eHealth interventions rely on a high standard of data security. This paper describes some of the key methodological, technical, and procedural issues that need to be considered to ensure that eHealth research and intervention delivery meet adequate security standards. The paper concludes by summarizing broad strategies for addressing the major security risks associated with eHealth interventions. These include involving information technology (IT) developers in all stages of the intervention process including its development, evaluation, and ongoing delivery; establishing a wide-ranging discourse about relevant security issues; and familiarizing researchers and providers with the security measures that must be instituted in order to protect the integrity of eHealth interventions.

## Introduction

Over the past decade, there has been a rapid growth in provision of online health interventions and the scientific evaluation of their efficacy and effectiveness [1]. A high standard of data security is critical to the overall success of any eHealth project whether it is concerned with scientific research or the provision of eHealth services. However, security considerations are typically an overlooked and underfunded aspect of the development, delivery, and evaluation of eHealth interventions. Moreover, the training of eHealth researchers rarely equips them to understand the key issues and challenges associated with online data security.

### Scope and Context

This paper is intended as a brief primer on data security for eHealth intervention researchers and providers. It aims to highlight the complexities and challenges associated with the security of eHealth interventions with the intention of better informing the activities of eHealth professionals who are not information technology (IT) specialists. It is not a comprehensive or systematic review of all security issues within the eHealth field and is not intended to provide specific risk mitigation solutions. Rather, it aims to highlight some key areas for consideration and to suggest measures that may help to address the major security risks of relevance to those commissioning and managing the development and delivery of interventions.

The discussion focuses on security considerations pertinent to *individual* software applications that are accessed by consumers for health prevention or treatment purposes either in research or “real-world” settings. Some of these security issues are illustrated with a focus on e-mental health interventions. This is for 2 reasons. First, the authors have many years of experience managing security issues in the e-mental health intervention domain both with respect to research and large-scale service provision. Secondly, security considerations are particularly critical in the domain of eHealth service provision and research due to the highly stigmatized nature of mental illness. The increasing popularity and availability of e-mental health interventions over the past decade has prompted psychological societies across the world to develop specialized guidelines for psychologists engaged in e-mental health activities [2-4]. These developments promote professional and ethical practices and are important for the protection of consumers of e-mental health interventions. However the realization of ethical standards is complicated in the realm of e-delivery. In particular, protection of consumer privacy and confidentiality, a central principle of professional psychological services, evolves into wide-ranging technical and nontechnical security considerations that need to be addressed.

### Definitions and Key Security Areas

**Figure 1:**
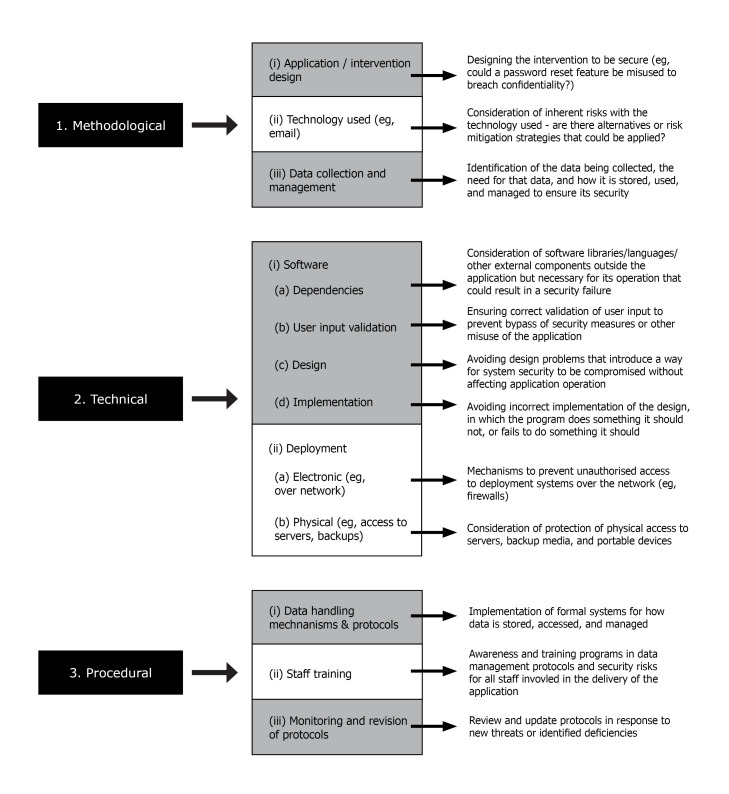
A summary of key security issues in the eHealth intervention environment

For the purposes of this paper, we use the term “security” to refer to the implementation of appropriate safeguards to protect user privacy and confidentiality. In the context of eHealth interventions, this means the appropriate collection and handling of user data, the protection of data from unauthorized access or modification, and the safe storage of data. Further, we distinguish between *methodological, technical,* and *procedural* security (see Figure 1). Methodological security is concerned with how the overall service is designed and what types of technology are used for which purposes. Technical security relates to how the software is developed and how it operates. Finally, procedural security refers to how the operators of the intervention handle and store collected data. Each category is separately examined in the next section.

There are no statistics on the overall prevalence of different types of security breaches in the domain of eHealth or e-mental health. However, a recent study of identity theft in the United States identified 115 reported breaches of security in the health care sector over a 3-year period [5]. Of these, 45% were classified as “hardware” problems whereby sensitive data stored on a physical device (eg, laptop or server) was compromised through unauthorized physical access to the device. A further 43% of breaches resulted from mishandling or misuse of data (including lost or stolen documents and media, processing errors, and incorrect disposal of data) and can be classified as procedural breaches. In total, 8% of the reported security breaches arose from intentional insider misconduct, and only 4% of the reported security breaches arose from the exploitation of a vulnerability in the application or system.

These findings emphasize the importance of taking steps to avoid hardware and procedural breaches in the deployment of eHealth interventions. However, clearly all areas of possible vulnerability must be addressed given the potentially high cost of even a single breach. Moreover, failure to address one category of vulnerability can render other security precautions useless. For example, a technical compromise that enables an attacker to gain remote access to an application database bypasses even the best measures to physically protect that hardware.

## Types of Security

### Methodological (Design) Security

Methodological security focuses on the risks associated with how an application is designed to operate, that is, which technologies are used and for what purpose. These issues must be considered as early as possible in an application’s development cycle since they are critical to much of the development process and impact on the required resources and timeline for the project.

Risk mitigation related to methodological security requires an understanding of the requirements and limitations of privacy protection in both intervention research and application development. For example, suppose an e-mental health intervention aims to facilitate online contact between a therapist and a client. In the first instance, the researcher or practitioner may conceptualize this as “email counseling.” However, email technology may not be the most appropriate communication method for this purpose since it is an inherently insecure medium. Although it is possible to institute precautionary measures such as email encryption, the operation and management of such measures pose significant challenges. For example, there are practical difficulties associated with securely swapping and storing encryption keys and the need to deal with multiple encryption standards [6]. In this situation, a more appropriate approach would be to use a secure, access controlled user environment within which the client can access and post messages from and to the therapist. Since the technological development process and associated procedural and technical security considerations are very different for each approach, it is critical that this is issue be addressed at the outset.

Another methodological security consideration involves deciding which data is collected and stored by the program and for what purpose. Data may be collected to facilitate the research and evaluation aims of the program and/or user information may be required for the software to operate as intended. In both cases, the collection and storage of data and access to these data need to be informed by ethical standards of research and health care delivery. The technical and procedural considerations that arise from the collection of these data will depend on their nature and purpose.

For example, suppose a user’s age is collected for the purpose of evaluating the relative effectiveness of an intervention in different population groups. Alternatively, a program may require information about the user’s age range in order to tailor the content to the user. A first consideration is whether a broad age range is sufficient for the intended purpose or whether more specific information is required. The more specific the information collected, the greater the possibility that an individual may be individually identifiable. This may be particularly true if the information is collected in the context of other personal data. Information that is not identifiable in isolation may be identiable when taken together with other data. The severity of a breach increases with the sensitivity of collected data. Thus, an important risk mitigation measure involves identifying the minimum level of detail required when collecting personal data. For example a user’s precise date of birth may be required in some circumstances. However, if the year of birth is sufficient, only this level of detail should be collected.

A further concern is the user’s role or potential to cause a confidentiality breach, for example, by losing or otherwise exposing their account details to others including those whom they may trust. For example, a person with access to a user’s email account may abuse a password-reset feature to gain access to an individual’s data on an e-mental health program thereby gaining access to sensitive personal information. Consideration should be given to the potential risks and ways in which the design or the information provided to users can mitigate these risks.

### Technical Security

The technical implementation and operational environment for an eHealth software application consists of many different IT and communication components. These include but are not limited to programming languages, databases, server hardware, data storage and backup systems, network switches, routers, and firewalls. Multiple IT specialties are involved in the management of this complex environment, including software engineering, system administration, database administration, and network engineering.

Although technical security considerations are complex and multifaceted, they can be broadly separated into 2 components: (1) the software application itself, and (2) the infrastructure (deployment environment) used to deliver it to end users. Each of these areas needs to be considered.

#### Software Application

It has long been acknowledged in the software engineering discipline that software defects arise not only as a result of coding errors, but also during the specification and design phases of a project. During the software development and testing phase, a common error is to focus solely on functional requirements (what the application will do) and ignore nonfunctional requirements such as the more difficult and specialized task of security testing [7,8]. Whereas functional testing focuses on ensuring the program does what it *is supposed to do*, security testing involves finding defects or flaws that allow an attacker to do something they are *not* *supposed to be able to do*. The latter is an inherently much more complicated challenge.

Consideration needs to be given to four common types of security failures [7]. These include (1) dependency insecurities and failures, (2) unanticipated user input, (3) design insecurities, and (4) implementation insecurities. Each of these is described below.

##### Dependency Insecurities and Failures

Dependency insecurities and failures are problems that occur when an external component that is used by an application contains a security vulnerability, or when an external component that provides security fails or becomes unavailable. In such cases, the application itself is secure, but a component it depends on is not, and this creates a security risk for the application. Most software, particularly Web applications, are dependent on a wide range of external components and applications in their delivery, and multiple examples of insecurity abound. As of January 2010, the US Government-operated National Vulnerability Database listed 40,260 unique vulnerabilities within operating systems, library functions, and applications that have been identified since the database began in 1999 [9]. Examples of particular relevance to eHealth interventions include database server vulnerabilities [10], Web server vulnerabilities [11,12], and faults within the programming language itself [13].

An insecurity with an external component does not necessarily mean the application can be or has been compromised. A vulnerability in a complex piece of software such as a database server may have no relevance to or impact on the application. It may be safe to delay the application of some fixes while ensuring others are urgently applied. What is important is that relevant IT expertise is used to make and act on such decisions.

All software applications depend on external components whether they are associated with the deployment of the application or are software components incorporated as part of the development of the application. In order to mitigate the risks associated with these components, it is essential that eHealth interventions are developed and deployed by IT experts with the knowledge and expertise necessary to manage issues associated with the application’s external components. This includes knowledge of each library or external component that has been introduced to the application environment (such as software libraries), and the expertise to identify and resolve any security problems associated with external dependencies. This in turn requires subscription to appropriate security bulletins that provide alerts about new potential issues; a competent evaluation of the impact of the vulnerability on the application itself particularly with respect to any implications it might have for user privacy and confidentiality; and testing and immediate deployment of relevant patches in situations that are deemed to pose a risk.

It is important that such IT security expertise is available throughout the life of any intervention and not only during the development phase. Although an application may meet security requirements when it is launched and first delivered, it may be identified as being subject to serious security risks over time. In such cases, the risk is not necessarily associated with any fault in the intervention software. Rather it may be due to the emergence of a newly identified security risk in an external component.

Dependency insecurities involving components directly used by applications are often less publicized than those involving components used in the deployment of applications (such as operating systems) since they are used by fewer applications. Clearly, however, they can be just as important. This is illlustrated by a flaw in commonly used bulletin board software that allowed a hacker to determine the password hash of a user, inject that into the log-in process, and log in as the user [14,15]. For applications that use such an affected component, the security flaw and available solutions (workarounds and installation of patches) need to be examined and a risk mitigation strategy adopted as a matter of urgency. This is particularly important since many hackers will specifically target unpatched security flaws once they are reported in the public domain.

Mitigating risks of dependencies that are associated with the *deployment* of the application (such as operating system or Web server flaws) are discussed as part of deployment environment considerations.

##### Unanticipated User Input Problems

Unanticipated user input problems arise where a software application does not correctly identify invalid or unexpected input or fails to correctly handle it. In the context of Web-based eHealth applications, this can compromise stored data, for example, through structured query language (SQL) injection and cross site scripting (XSS) attacks [16,17]. SQL injection [16] enables an attacker to modify database commands and potentially execute completely different commands. This can lead to a data breach since checks and constraints that control access to the stored datasets can be removed. XSS attacks [16] involve insertion of additional code into a Web page that is viewed by others. This could occur for example on a bulletin board where a user writes a post that includes malicious hypertext markup language (HTML) code. When this post is displayed to other users, the code may trigger a number of undesirable and potentially serious consequences, such as embedding content from a malicious website or reporting the user’s session information back to an unauthorized server. The potential impact of these kinds of attacks on the protection and integrity of user data cannot be understated. Fortunately, with appropriate software development, these risks can be (relatively easily) mitigated.

Each of these problems occurs as a result of an application failing to correctly “escape” control characters in the input (eg, quotation marks, semicolons, or html tags) that when inserted in a different context (eg, an SQL statement or an html page) have a different meaning and are executed differently. Input provided by a user should always be assumed to be potentially hostile, and any input that could represent code in a different context should be escaped or removed. More generally, software should always validate input data [18,19]. Validation should occur on length (whether too long or too short) and type (eg, integer or string), and input should be examined on syntax or range as appropriate (eg, does it match the format of an email address or a postal code). The Open Web Application Security Project advocates 3 data validation approaches: acceptance of only known data, which are validated against a white list of known “good” values; rejection of data known to be problematic, such as input containing invalid data; or sanitization of problematic data into an acceptable format [18].

All eHealth interventions should be developed to validate input data and deal with invalid data appropriately. This is an essential aspect of protecting user confidentiality and privacy that should be routinely considered as part of any application development.

##### Design Insecurities

Design insecurities are flaws that are introduced at the design stage. These are oversights or failures that are inadvertently designed into the application. Design flaws are often not detected during normal testing. Such flaws can be quite simple, such as not observing that 2 systems communicate confidential data in an unencrypted form over the Internet. Often, however, they are more complicated. For example, in July 2008 the United States Computer Emergency Readiness Team identified a flaw in the way domain name system (DNS) servers verify responses that could allow an attacker to introduce fake entries such that a user would be sent to an incorrect server designed to steal confidential information or install malicious software [20]. The vulnerability relied on the attacker being able to set up his or her own DNS server and apply clever timing in order to exploit it. This flaw posed a risk with serious privacy implications for affected users.

The logical approach to mitigating design insecurities is to reduce the risk that they occur in the first place, particularly given that design flaws are difficult to detect in testing and that the earlier they are found, the less expensive they are likely to be to fix [21]. Such analysis of design is a knowledge intensive process [22]. Therefore the best approach for mitigating design flaws in eHealth interventions is to ensure that they are developed by IT specialists who have a good understanding of possible design risks within the context of eHealth. Those commissioning the development of the interventions should be aware of the need to employ specialists who can apply such approaches in the design process. Examples include Microsoft’s STRIDE approach [23] (STRIDE is an acronym for spoofing, tampering, repudiation, information disclosure, denial of service, and elevation of privilege), which applies threat analysis to each component of the system model and Verdon’s risk analysis process model, which is applied at the design phase [22]. Moreover, it is important to ensure that IT staff employed on an eHealth project stay informed of design flaws that are reported in other applications, such as through the publication in Common Vulnerabilities and Exposures [24].

##### Implementation Insecurities

Implementation insecurities are bugs in the coding whereby the application *does not correctly do something that it is designed to do* (such as performing a validation check on a certain type of input data) or where it *does something that was not intended* (such as creating a temporary file containing confidential information on the server without appropriately securing access permissions). These bugs may unintentionally disable or compromise security measures that were part of the application’s design.

The employment of programmers who use appropriate software development methodologies can minimize the frequency of defects occurring in the first instance. Nevertheless, testing for implementation errors is a core element of any software development life cycle [25]. It establishes whether a coded piece of software operates as it was intended. This includes whether it protects user privacy and confidentiality. Testing processes need to be planned, efficient, and systematic to ensure bugs are detected and rectified within time and resource constraints. The testing must involve both those who commissioned the eHealth intervention and those responsible for the IT development of the software. Accordingly, researchers and providers must allocate sufficient time and resources to the iterative testing phases as part of the development process.

#### Deployment Environment

The deployment environment refers to how the software application is delivered—including electronic aspects (such as server operating systems and access controls) and physical aspects (such as physical location of the servers themselves). Security considerations within the deployment environment include the protection of stored data from *electronic threats* (such as unauthorized electronic access to servers or a vulnerability in an operating system) and *physical threats* (such as those that result from unauthorized physical access to a server).

As previously noted, a privacy breach associated with the electronic compromise of systems is uncommon; however, when it occurs it can have a major impact. Electronic risks can be mitigated by assigning staff with appropriate skills to the task of monitoring and managing deployment systems throughout the life of the application. This will include safeguarding against the security risks associated with external dependencies.

Physical hardware breaches occur more frequently despite the fact that they can be prevented readily. This suggests that, in practice, protection against such threats is often not appropriately prioritized. In the deployment environment, protecting against physical hardware risks involves preventing unauthorized physical access to servers and the use of backup media and other data storage devices.

Often organizations providing small-scale eHealth interventions do not directly manage the deployment environment used to deliver their programs or are responsible for only a small section of it. This is not necessarily undesirable since it may provide the eHealth provider with access to specialist skills and existing infrastructure. For example, eHealth teams based at universities may rely on their university’s general IT infrastructure and staff, and small research organizations may employ professional hosting providers to deliver their programs. However, the reliance on external others does not mean that providers can ignore the security risks associated with this key component of the application’s delivery.

All eHealth intervention providers should inform themselves of the types of practices that contribute to a secure deployment environment so that they can ensure that implemented strategies are commensurate with their expectations of user privacy and confidentiality protection. Providers should be aware of the common strategy of “defense in depth,” that is, multiple layers of security should be implemented to ensure that a failure at one point does not compromise the system. For example, a firewall implemented on a server would guard against the failure of a firewall higher up in the network topology.

Table 1 provides a set of questions and topics that should be discussed with whoever is responsible for the deployment environment. It is not an exhaustive list, and it may be most appropriately undertaken in the context of a broader risk management analysis. However, it provides a starting point for researchers or developers who have limited knowledge of the issues of application delivery and addresses the major threats to confidentiality identified by others in the field of public health e-implementation [26]. Obviously, the inclusion of technical staff within the research and development team will increase the specificity of the discussed safeguards, but even without this expertise, intervention providers should take responsibility for ensuring that basic measures are in place to protect the data they collect.

**Table 1:** Examples of questions that are relevant to the deployment environment

Area	Question	What it Means
Data: servers	Where are the servers located?	Knowledge of all physical locations where the data will reside, including off-site or redundant systems
	What are the physical protection measures for the servers?	Knowledge of the strength of physical access controls such as door locks/access cards, locks in server racks, monitored closed circuit television
	Who has access to the servers? How is this access controlled and monitored?	Knowledge of who has physical and electronic access to servers, who grants this access, and how access is revoked when no longer needed or at the end of a staff member’s employment
	What is the disposal policy for old or failed hardware?	Knowledge of how old drives and media are secured is necessary. Appropriate measures include secure erasure and physical destruction.
	What remote access is there to data on the servers, and who has access to these data?	Identification of the means by which data can be remotely accessed, such as over file shares or use of remote access utilities, and who has access to these
Data: backups	Where are backups stored?	Knowledge of all physical locations where backup media will be stored, including off-site locations
	How are backups transported to storage?	If backup storage is physically separate from servers, ensuring that backups are safely transported to storage locations
	Who has access to the backups? How is this access controlled and monitored?	Identification of who can access the backups, who grants this access and how it is revoked when no longer needed or at the end of a staff member’s employment
	Is encryption used? Who has access to the keys/passwords?	Encryption reduces the risk of a breach if media are lost, stolen, or disposed of incorrectly. If there is no encryption, safe physical storage becomes even more critical.
	What is the disposal policy for old/failed media?	Knowledge of how old backup media are secured is necessary. Appropriate measures include secure erasure and physical destruction.
Servers	Are server operating systems and software updated with required security patches?	Ensuring that there is a mechanism or policy in place whereby security patches are applied to servers and supporting software within an appropriate time frame
Network security	Are firewalls in use on the network, how and where?	Firewalls filter unwanted and potentially malicious traffic. Multiple layers of firewalls reduce the risk of internal attacks.
	Are mechanisms in place for intrusion detection?	Ensuring that an attack or potential attack can be identified enabling it to be prevented or handled quickly
Policies	What security policies, protocols, and processes are in place?	Establishing that a formal security policy has been adopted and that risk mitigation is a high priority
	How are security policies monitored and enforced?	Ensuring that security protocols are actively implemented is necessary. A policy or risk mitigation strategy needs to be applied in practice to be useful.

### Procedural Security

Procedural security considerations concern the internal processes and mechanisms surrounding data handling. This includes who handles which data in which situations, what they do with the data, and appropriate procedures for handling a breach should it occur.

All eHealth application data is collected either through direct input by the user or through other communication mechanisms such as email, instant messaging, or telephone. All forms of potentially identifying data, including clinical notes or electronic communications with users must be appropriately handled. An intervention that has high standards of technical security quickly becomes vulnerable to privacy breaches if staff act inappropriately. Common examples of procedural failures include loss or theft of an external hard drive that contains insecure data or copying of data into insecure locations such as shared drives that can be accessed by others.

In order to protect user confidentiality and privacy, e-mental health and eHealth intervention providers need to implement comprehensive data security protocols for staff involved in the operation and deployment of the application. This may be undertaken in the context of the development of a staff security awareness and training program. Guidelines for developing such a program, together with details of helpful awareness and training resources, have been published by the US National Institute of Standards and Technology [27,28]. The importance of these measures cannot be understated, since the vast majority of health care e-privacy breaches occur as a result of procedural failures, including those caused by individuals failing to protect data stored on physically portable hardware [29]. Clearly, procedural security protocols need to be developed in the context of the ethical standards associated with provision of the intervention. However, they must also be informed by an understanding of the human factor risks that arise when staff use particular technologies.

Protocols need to be effectively implemented so that all staff and students, regardless of the size of the eHealth organization in which they are operating, understand how to deal with and protect sensitive data. Routine and ongoing monitoring of procedural risks is also required so that protocols can be adapted as required to rectify deficiencies in the existing measures and respond to new threats. Some aspects of the protocol will be specific to the application that is being delivered; others will address more general issues. As a starting point, Table 2 lists some of the most important procedural areas for consideration in eHealth intervention delivery. Again, this table is not intended to provide a comprehensive guide to procedural issues. However, it will provide the reader who has little background in procedural security issues some key issues to consider when developing protocols to protect data.

**Table 2:** Examples of questions and actions that are relevant to procedural security

Area	Question/Action	What it Means
Data	Are appropriate access controls applied to data?	Ensuring appropriate permissions and access controls are applied to files on network shares and any other access controls as relevant
	Encryption of portable devices such as laptops, external hard drives, and USB keys	These devices may be lost, stolen, or misplaced and thus represent a potentially significant threat to data security. If there is a chance these devices could be used to store confidential information (or data which could enable reidentification of information), then they should be encrypted. Applications such as TrueCrypt (http://www.truecrypt.org/) can support this.
	Encryption of desktop computers, if appropriate	Encrypting systems that come into contact with confidential information reduces the risk of a breach in the event of theft or incorrect disposal.
	Is identifying information really stored separately from the data?	Electronic storage means it can be difficult to physically separate identifying information from deidentified data. At the very least, electronic access controls should be set up to ensure virtual separation. It may be appropriate for a data manager to manage these access controls.
	Storage of email correspondence and other electronic records in encrypted environments	Confidential records should be stored in an encrypted format.
	Is data transferred between staff or collaborators? Under what circumstances and how is the transfer undertaken?	Staff need to understand the security risks of communication technologies such as email, and procedures needed to be implemented to address these risks in different situations. For example, email addresses and other identifying information need to be removed if forwarding user emails for discussion with colleagues, and restricted access environments should be used for transfer of data sets.
Hardware	Is there a policy and procedure in place for the disposal of old hardware and media?	Old computers and media should be securely erased before they are sold or recycled. If appropriate, storage devices should be physically destroyed.
Policy	Have processes been established for handling a breach if it were to occur?	Any breach needs to be handed effectively—knowing the steps that need to be taken is vital. This should include both steps for handling the breach itself and necessary review and rectification of processes to avoid future breaches.
	Has a policy been developed which addresses the risks of relevant technologies (email, external drives, remote access, etc)?	The tools available to an organization can pose substantial security risks if used inappropriately, but staff without an IT/security background may be unaware of this. Relevant risks need to be assessed and strategies/tools put in place to assist in mitigating them.
	Has a policy for handling staff turnover been developed?	Departure of staff from the organization needs to be handled suitably, including the return of any hardware (PDAs, external drives, laptops, etc), any documents that may be stored remotely (confidential or otherwise), and, if appropriate, the sanitization of computers.
	Are new and existing staff educated about security risks and trained to implement privacy measures?	Policies need to be communicated to all staff, including reminders on a regular basis. If security is not part of day-to-day operations, then a breach may be more likely to occur. Staff need to understand how to appropriately apply security policies in their field of work.
	Is there regular review and monitoring of relevant policies and their application?	Policies and associated outcomes need to be enforced and reviewed regularly so that they can be modified as required.

## Overall Strategies: The Present and the Future

Designers and providers of eHealth interventions need to be aware of and mitigate the complex security risks associated with delivery of their applications. Ongoing risk assessment should be conducted in all of the above areas, and appropriate mechanisms, including a security protocol, must be put in place to guard against breaches [29]. In order to facilitate this process, 2 broad strategies can be used: (1) appropriate use and integration of IT staff in all stages of the project and (2) engagement in a wide-ranging discourse about security issues in eHealth interventions.

### Appropriate Use of IT Expertise Across the Project Life Cycle

As discussed above, specialist IT staff need to be consulted at all project stages of the eHealth intervention research, development, and delivery to ensure privacy and confidentiality requirements are translated into practice. The security of eHealth interventions requires an understanding of the ethical obligations of the health or psychological service and, more specifically, knowledge of how the use of different technologies can impact on meeting these obligations. There are 2 ways of meeting this challenge: by fostering the development of transdisciplinary internal experts or by relying on external expertise that is managed internally within an overall risk mitigation context.

In-house transspecialist staff can facilitate the proper consideration of methodological (design) security at an early stage of the project, manage or deliver technical measures, and contribute to the development and implementation of risk mitigation procedures relevant to the project. The use of multidisciplinary in-house teams also builds a knowledge base of which methodologies and processes work most effectively for that organization’s eHealth research or delivery program [30].

If appropriate IT staff cannot be included as part of the project team, it falls to the researcher or eHealth intervention provider to ascertain that appropriate security considerations are undertaken by whoever is responsible for the development and the delivery of the program respectively and to ensure that there are provisions for ongoing management of these considerations.

Appropriate involvement of IT staff necessarily requires that budgets and timelines reflect the requirement for specialist IT expertise and processes that ensure the secure delivery of interventions, not just as part of the software development phase, but for the life of the application. Many eHealth projects do not currently progress from the evaluation stage (where their provision to study participants is evaluated) to wider availability to consumers [31]. There are many reasons for this, only some of them involving IT considerations. Nevertheless, a necessary condition for the widespread delivery of eHealth programs to the public is the involvement of suitable IT expertise to ensure the appropriate planning, design, and delivery of secure and scalable applications.

### An eHealth Intervention Security Discourse: The Future

Within a rapidly changing technology environment, eHealth interventions and e-mental health interventions in particular have gained acceptance. Although the potential of these interventions is huge, they introduce a whole new set of risks to traditional health and mental health service delivery. A wide-ranging discussion is needed to enhance stakeholder understanding of the mitigation of these risks, both with respect to the e-domain in general, and the eHealth and e-mental health service types in particular. Enhanced discourse about security should include reporting of emergent risks, mitigation approaches, and breaches. Holistic or specific security measures that are introduced as part of an application’s design, delivery, or evaluation need to be reported across disciplines. Privacy breaches, if they occur, should be examined in appropriate technical or procedural detail so that they can serve as a shared repository of knowledge designed to ensure that mistakes are avoided in the future. Shared discourse about strategies for coping with potential threats is also important to the development of best practices in the eHealth research and delivery domain.

To our knowledge, there are no comprehensive agreed upon standards for security in e-mental health or eHealth interventions and research specifically. However, consumer confidence in electronic health measures requires assurance and demonstration of appropriate security measures [32], and privacy and confidentiality is particularly important to consumers of e-mental health interventions. There are legislated obligations surrounding the collection and use of sensitive personal information in different legislative contexts (eg, American Health Insurance Portability and Accountability Act in the United States). However, the eHealth field needs to create standards that not only meet such requirements, but also provide the highest standards of health and mental health intervention. Useful standards that have a high applicability to the challenges of eHealth and e-mental health interventions can only be created through collaborative exploration and debate by those involved in the provision of these programs.

## Conclusion

The research and development of eHealth interventions and e-mental health interventions has expanded rapidly over the last decade as the potential public health impact of innovative e-mental health delivery techniques have been demonstrated [33]. Increasingly, such interventions are being implemented in practice. However, creating an adequate, scalable, and secure eHealth intervention or e-mental health intervention requires more than a good idea, a budget, and the name of an IT company that is able to build a specified program for the allocated resources. The challenge for the developers and providers of such services is to set and meet security standards of delivery that ensure that consumers can use these services safely and with confidence. 

## Abbreviations

IT: information technology
XSS: cross site scripting
DNS: domain name system
HTML: hypertext markup language
SQL: structured query language
